# Social support, social anxiety, psychological resilience, and antisocial behavior in sports among college students: a cross-sectional study

**DOI:** 10.3389/fpsyg.2025.1656847

**Published:** 2025-09-02

**Authors:** Xi Chen, Yi Li, Junkai Zheng

**Affiliations:** ^1^School of Sports Science, Guangzhou College of Applied Science and Technology, Guangzhou, China; ^2^School of Economics and Management, Guangzhou College of Applied Science and Technology, Guangzhou, China

**Keywords:** social support, antisocial behavior in sports, social anxiety, psychological resilience, college student

## Abstract

**Introduction:**

This study explored the relationship between antisocial behavior in sports among college students and social support, as well as the mediating mechanisms of social anxiety and psychological resilience on antisocial behavior in sports.

**Methods:**

Using a simple random sampling survey method, we collected data from 1,421 college students aged 18-24 (female = 604, 42.51%) in Guangzhou and Zhaoqing, China. The Perceived Social Support Scale (PSSS), the Prosocial and Antisocial Behavior in Sport Scale (PABSS), the Liebowitz Social Anxiety Scale (LSAS), and the Connor-Davidson Resilience Scale (CD-RlSC) were utilized.

**Results:**

The research demonstrates that social support negatively predicts antisocial behaviors in sports (*β* = −0.108, *p* < 0.05), with social anxiety serving as a significant mediator (*β* = −0.096, *p* < 0.05). Psychological resilience moderates three key relationships: between social support and social anxiety (*β* = −0.237, *p* < 0.05); between social anxiety and antisocial behavior (*β* = 0.173, *p* < 0.05); and between social support and antisocial behavior(*β* = −0.198, *p* < 0.05).

**Discussion:**

To bolster social support, an integrated tripartite supportnetwork (athlete-coach-psychological coach) is recommended. Mechanisms such as structured event retrospectives may enhance psychological resilience, whereas mindfulness training and cognitive restructuring interventions could target social anxiety reduction, thereby potentially attenuating antisocial behaviors.

## 1 Introduction

Antisocial behavior in sports refers to actions that are harmful or detrimental to others, including verbal abuse of teammates and threats or intimidation directed at opponents (Ian et al., 2020). Such behavior causes tangible harm, as studies have linked athlete violence to psychological distress, post-traumatic stress disorder (PTSD), and dropout rates (Anouk et al., 2018). Research on antisocial behavior in sports more than an academic exercise—it is a public health imperative and social responsibility. Within this framework, identifying and understanding the key antecedent variables that effectively predict these behaviors has become a focal point in psychology research (Wenchao et al., 2020).

Social support is defined as the network of individuals or groups that provide emotional and practical assistance to an individual, thereby reducing psychological distress, enhancing social adaptability, and promoting mental well-being (Wenchao et al., 2020). This concept can be categorized into two primary types: (1) actual support (the tangible assistance received) and (2) perceived support (an individual’s subjective evaluation of available support). Mark et al. (2014) introduced a multidimensional model of perceived social support, later applied to sports contexts (Alyson et al., 2021). The model comprises three key components: firstly, the cognitive dimension, which mitigates an individual’s awareness and assessment of the dangers posed by stressful situations; secondly, the emotional dimension, which alleviates fear and anxiety triggered by stress; and thirdly, the behavioral dimension, which aims to reduce both physiological and logical reactions, as well as inappropriate behaviors that may arise from stress. Through this framework, a strong relationship emerges between social support and sports behavior. This external assistance plays an irreplaceable key role in ensuring that individuals have the material conditions and knowledge reserves they need (Kathleen et al., 2021). Moreover, it conveys positive emotions and values that help reduce antisocial behavior in sports (Mark et al., 2017). These actions strengthen group cohesion and interpersonal bonds, effectively reducing antisocial behaviors like blaming teammates or verbally abusing opponents (Martin et al., 2016). Through previous studies, we propose:

*Hypothesis 1*: social support negatively predicts antisocial behavior in sports.

Social anxiety is characterized by persistent nervousness and withdrawal in social settings due to a fear of judgment or embarrassment (Preeti and Narendra Nath, 2021). It is marked by intense worry, tension, or fear (Nathaniel et al., 2023). As an internalized issue that arises during social interactions, social anxiety significantly influences the study of individual interpersonal behavior (Rubén et al., 2020). Research shows that the social support that individuals feel can often act as a protective buffer, effectively reducing social anxiety and related psychological distress (Marie et al., 2022). Individuals who feel highly supported by people around them and their social networks are often able to get more positive motivation from it, which helps to reduce the tension in the social environment and significantly reduces the frequency of social anxiety (Liu et al., 2023). Recent studies have demonstrated that social anxiety not only significantly elevates perceived stress levels and state anxiety in exercise contexts (Tamura et al., 2022), but also triggers typical patterns of social avoidance behavior. This psychological state is frequently accompanied by various negative behavioral manifestations; on one hand, it increases the frequency of antisocial behaviors in sports, such as verbal aggression and physical conflicts (Bruner et al., 2018; Forsdyke et al., 2022). Prior research indicates that individuals enjoying strong social support typically exhibit lower social anxiety. Furthermore, the degree of social anxiety influences motor imagery; elevated social anxiety correlates with a higher incidence of antisocial behavior in sports. Consequently, we propose:

*Hypothesis 2*: Social anxiety mediates the association between social support and antisocial behavior in sport.

While deficits in social support and social anxiety may contribute to antisocial behavior in sports, these factors do not fully account for all instances. A more comprehensive examination is required to identify the mediating mechanisms underlying these relationships. The social support buffering hypothesis posits that interpersonal resources can mitigate the negative psychological and psychological and physiological impacts of stress, the extent to which adverse outcomes are effectively mitigated (Marie-Christine et al., 2014). In the study of exercise behavior, this hypothesis is frequently utilized to explain how social support enhances individual participation in, or maintenance of, regular exercise by regulating psychological, social, and environmental factors (Thomas et al., 2009). Within sports activities, family members, peers, and educators serve as critical sources of psychological support, where individual coping capacity emerges as a key determinant of adaptive functioning (David and Mustafa, 2012). Psychological resilience-conceptualized as an adaptive capacity, refers to the dynamic cognitive-behavioral process through which individuals adjust their functioning to achieve optimal alignment with changing environmental demands (Allison et al., 2023). This adaptive capacity enables individuals to maintain psychological well-being despite adverse events (William et al., 2020). Research has shown that when individuals face changes in their external environment, social support significantly enhances psychological resilience, boosts self-confidence, and reduces perceptions of stress and negative emotional responses (Yiqing and Sonia, 2024). That individuals’ psychological resilience can play a significant buffering role when facing negative interpersonal interactions (Ömer, 2024). Maintaining calmness during interpersonal difficulties, managing emotions, and alleviating anxiety and distress are defining characteristics of robust psychological resilience (Lisa et al., 2017; Mpaphi et al., 2021). Psychological resilience enhances the processes of social support, social contact. Psychological resilience can be improved through various methods, including stress buffering, self-efficacy enhancement, and emotion regulation, thereby reducing social anxiety (Sandra et al., 2016). The alleviation effect of social anxiety can be achieved through the intervention function of psychological resilience, thereby facilitating active communication with teammates, tolerance for teammates, respect for opponents, and other prosocial behaviors (Lisa et al., 2017; Minghui et al., 2018). Within the theoretical framework of psychological resilience and emotion regulation, psychological resilience adjustment strategies can be used to increase or reduce the negative or positive impact of goals, and situational strategies can enhance the process of social support, social contact and intimacy, effectively reduce social anxiety and increase prosocial behavior (Allison et al., 2023). Based on this theoretical framework, we propose:

*Hypothesis 3*: Psychological resilience serves as a moderator of the association between social support and social anxiety.

*Hypothesis 4*: Psychological resilience serves as a moderator of the association between social anxiety and antisocial behavior in sports.

*Hypothesis 5*: Psychological resilience serves as a moderator of the association between social support and antisocial behavior in sports.

**Figure 1:**
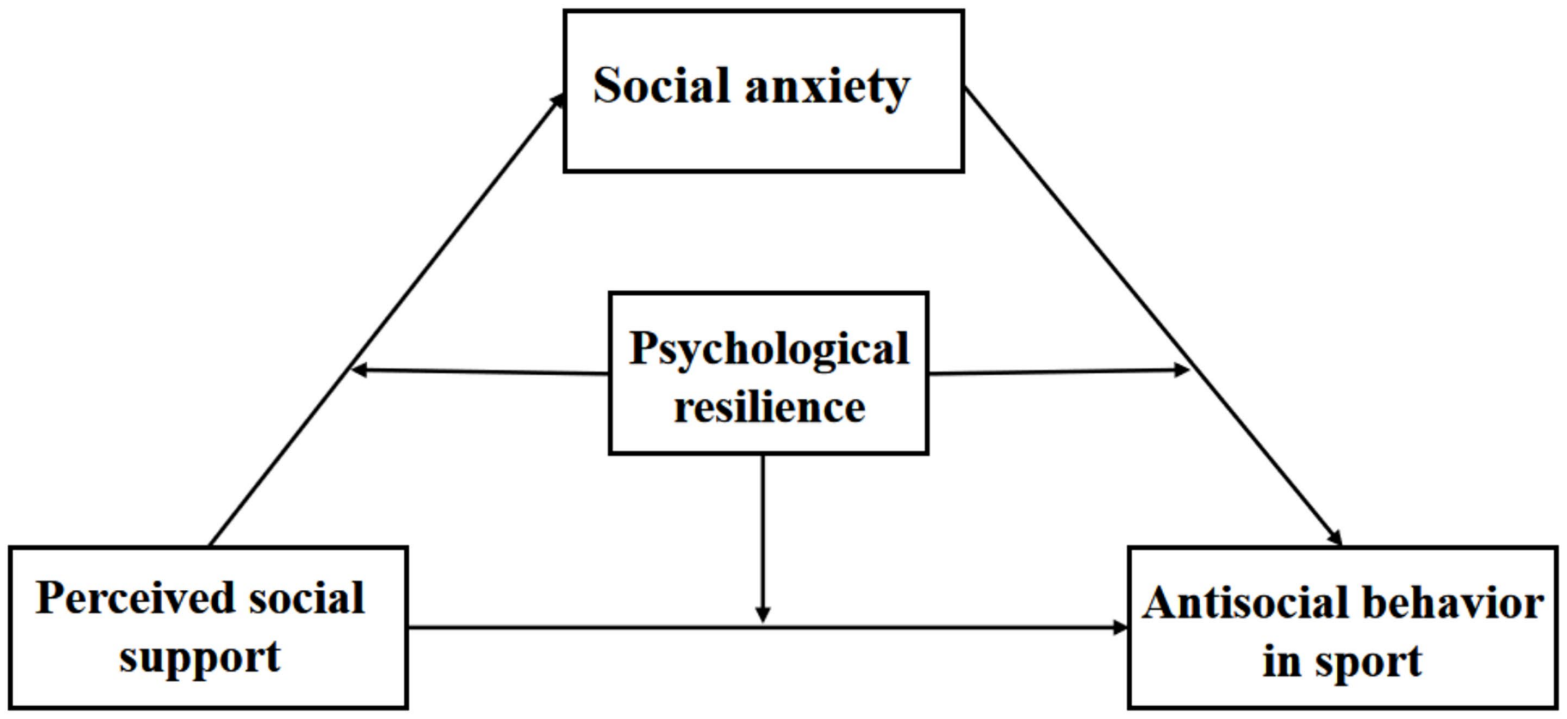
Moderated mediation model.

Research confirms that social support serves as a vital mechanism for fostering social adaptation and improving mental health. Nevertheless, systematic empirical investigations into its differential effects on antisocial behaviors in competitive sports remain scarce. This study examines the dual nature of social support’s influence (both facilitative and inhibitory) on such behaviors, while also investigating how social anxiety mediates the relationship between social support and sports-related social conduct. Additionally, Explores psychological resilience’s moderating role. These findings offer scientific foundations for college sports education, student mental health interventions, and athletic club management. Based on these outcomes, we propose a “regulatory-mediational” model (see Figure 1).

## 2 Methods

### 2.1 Participants

A convenience sampling approach was adopted, with data collected via the Questionstar (2024) platform. Between March and May 2024, questionnaires were administered to 1,287 undergraduate students (ages 18–22) from eight colleges in Guangzhou and Zhaoqing. Participants included both genders, with all having engaged in regular sports activities—either organized or self-directed—at least once weekly for the past 6 months. Exclusion criteria comprised severe physical illnesses (e.g., heart disease) or sports-related contraindications that might restrict participation. Before commencing data collection, participants received comprehensive details regarding the study’s objectives and assurances concerning the confidentiality of their data.

The final sample, determined after applying the inclusion criteria, included 1,421 valid responses, producing an effective response rate of 89.9% (Table 1). This high level of response underscores the reliability of the collected data and strengthens the overall findings of the study. The sample comprised 817 male (57.49%) and 604 female students (42.51%), with a mean age of 20.49 years (SD = 0.647). Age distribution of the tested students was as follows: 131 students aged 18 (9.22%), 275 students aged 19 (19.36%), 373 students aged 20 (26.25%), 300 students aged 21 (21.11%), 143 students aged 22 (10.06%), 143 students aged 23 (10.06%), and 56 students aged 24 (3.94%). Academic year distribution was: Freshmen: 29.49% (*n* = 419), Sophomores: 25.48% (*n* = 362), Juniors: 25.33% (*n* = 360), and Seniors: 19.70% (*n* = 280).

**Table 1:** Demographics for college students (*n* = 1,421).

Characteristic	*n*	Percentage in sample
Sex		
Male	817	57.49%
Female	604	42.51%
Age		
18	131	9.22%
19	275	19.36%
20	373	26.25%
21	300	21.11%
22	143	10.06%
23	143	10.06%
24	56	3.94%
Academic year		
Freshmen	419	29.49%
Sophomores	362	25.48%
Juniors	360	25.33%
Seniors	280	19.70%

### 2.2 Test tool

#### 2.2.1 Social support

The Perceived social support scale (PSSS) adapted into Chinese by Qianjing (2001), measures three dimensions of social support: family support, friend support, and other support. With demonstrated high reliability (Cronbach’ s *α* = 0.94) and validity, it has become a widely adopted tool for assessing perceived social support in Chinese populations, particularly among college students (Yitong and Yangqiang, 2023).

#### 2.2.2 Social anxiety

The Liebowitz Social Anxiety Scale (LSAS), revised by Chunzi et al. (2004), is a 15-item instrument assessing social anxiety symptoms. Utilizing reverse scoring methodology, higher total scores indicate greater social anxiety severity. Psychometric evaluations have demonstrated strong reliability (Cronbach’s *α* = 0.87) and validity within Chinese student populations (Guanghai et al., 2023).

#### 2.2.3 Psychological resilience

The Connor-Davidson Resilience Scale (CD-RISC), revised by Yun et al. (2016), is a 25-item instrument. It employs a 5-point Likert scale (ranging from 1 “strongly disagree” to 5 “strongly agree”), with both positively and negatively worded items. Higher scores indicate greater psychological resilience. Psychometric evaluations have demonstrated good reliability (Cronbach’s *α* = 0.83) and validity among Chinese student populations (Lianhua et al., 2022).

#### 2.2.4 Antisocial behavior in sport

The prosocial and Antisocial Behavior in Sport Scale (PABSS), revised by Dapeng (2012), is a 23-item instrument consisting of two subscales: 8 items measuring prosocial behavior and 15 items assessing antisocial behavior. Scoring follows a directional interpretation, where higher scores on each subscale indicate greater tendencies toward the respective behaviors in sports contexts. Psychometric evaluations have demonstrated strong reliability (Cronbach’ s *α* = 0.88) and validity for assessing moral behaviors among Chinese collegiate athletes (Dapeng and Cangzhu, 2022).

### 2.3 Data analysis

Statistical analyses (descriptive statistics and Pearson correlation) were performed using SPSS 21.0 (IBM, 2024). For assessing the mediation role of social anxiety, we employed the Sobel test along with bootstrap confidence interval analyses (5,000 resamples) using Mplus (2024). Structural equation modeling (SEM) was employed to assess the moderating role of psychological resilience. Simple slope analyses were performed at one standard deviation above and below the moderator’s mean. Johnson-Neyman plots were generated to visualize the conditional effects across the range of the moderator.

## 3 Results

### 3.1 Descriptive statistics of each variable

**Figure 2:**
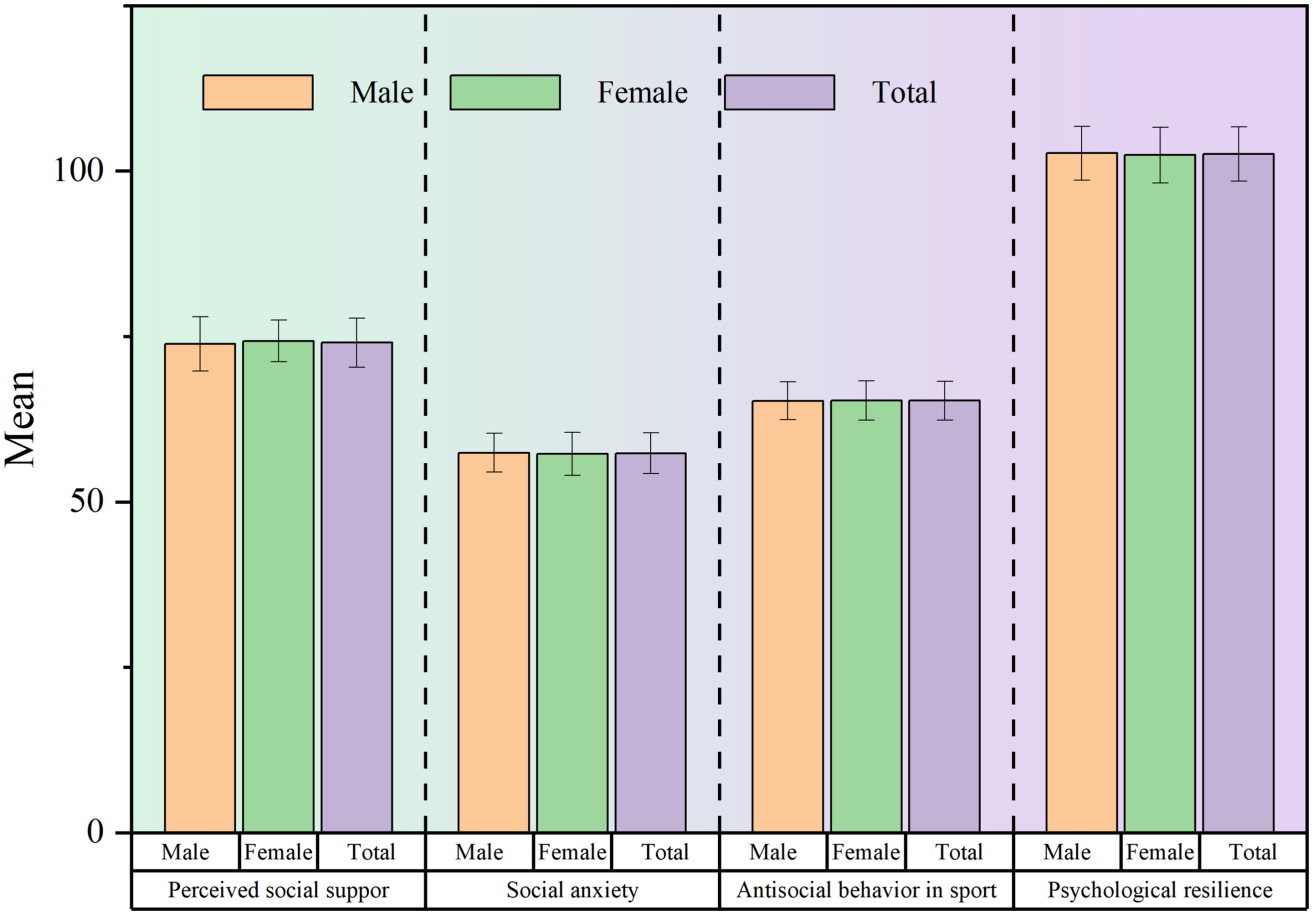
Descriptive statistics of each variable.

Descriptive statistics for key variables are illustrated in Figure 2. Overall, participants reported moderate levels of perceived social support (*M* = 70.4, *SD* = 4.082), with females (*M* = 74.3, *SD* = 3.139) scoring slightly higher than males (*M* = 73.85, *SD* = 4.082). Social anxiety levels were found to be in the mid-range (*M* = 57.34, *SD* = 3.084), with males exhibiting higher anxiety (*M* = 57.42, *SD* = 2.949) compared to females (*M* = 57.23, *SD* = 3.249). Regarding antisocial behavior in sports, the total sample mean was *M* = 65.28 (*SD* = 2.912), showing minimal gender differences (males: *M* = 65.27, *SD* = 2.874; females: *M* = 65.29, *SD* = 2.964). Psychological resilience emerged as the highest-scoring construct (*M* = 102.57, *SD* = 4.135), particularly among male athletes (*M* = 102.68, *SD* = 4.075) compared to females (*M* = 102.41, *SD* = 4.214).

### 3.2 Correlation analysis of each variable

**Figure 3:**
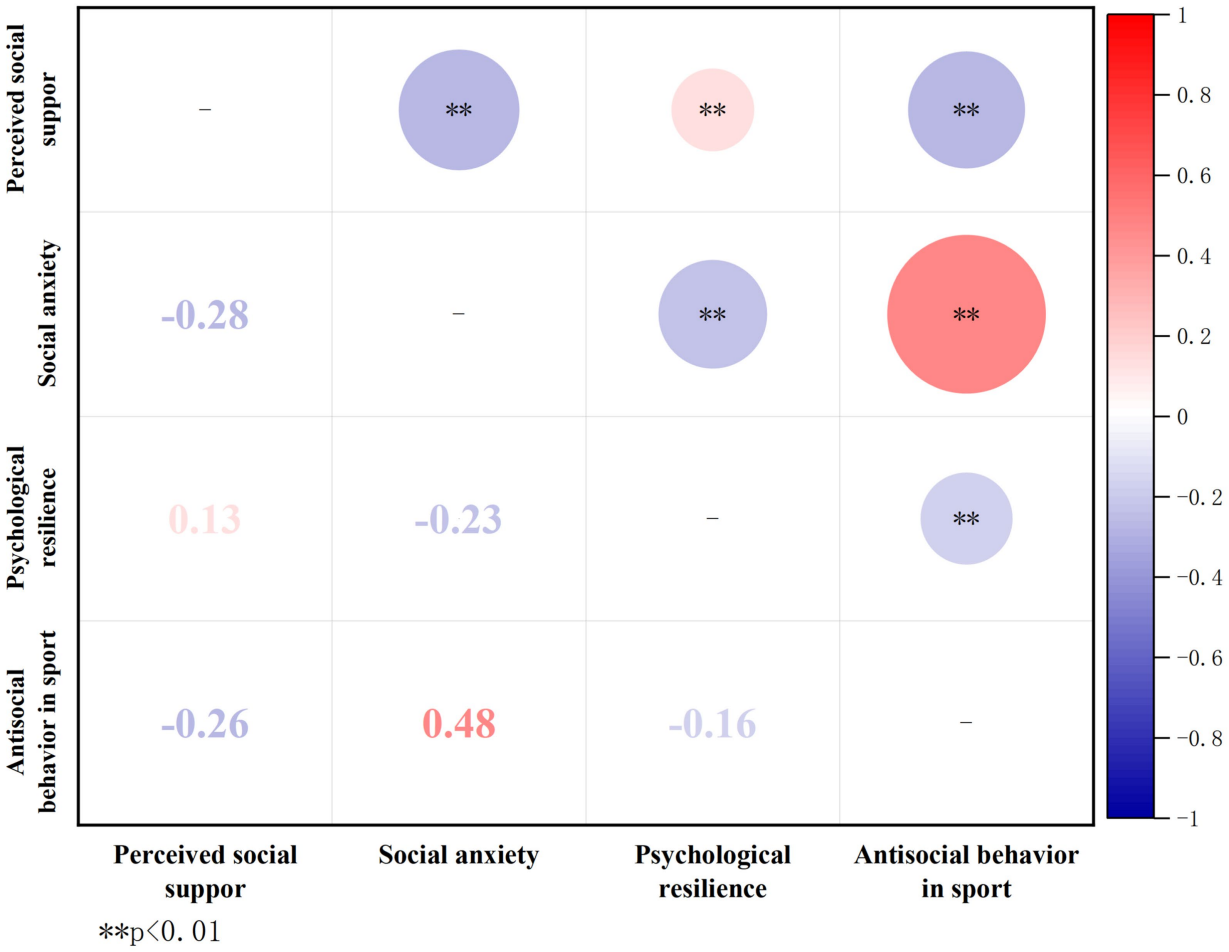
Correlation heatmap of variables.

Pearson’s correlation statistics were utilized to perform an in-depth analysis of the connections between perceived social support, social anxiety, psychological resilience, and antisocial behavior in sports (see Figure 3). Significant positive correlations were found between perceived social support and psychological resilience (*r* = 0.13, *p* < 0.01), as well as negative correlations with social anxiety (*r* = −0.28, *p* < 0.01) and antisocial behavior in sports (*r* = −0.26, *p* < 0.01). Psychological resilience shows a negative correlation with both social anxiety (*r* = −0.23, *p* < 0.01) and antisocial behavior in sports (*r* = −0.16, *p* < 0.01). Notably, social anxiety displays a positive relationship with antisocial behavior in the context of sports (*r* = 0.48, *p* < 0.01).

### 3.3 Social anxiety mediating effect

**Figure 4:**
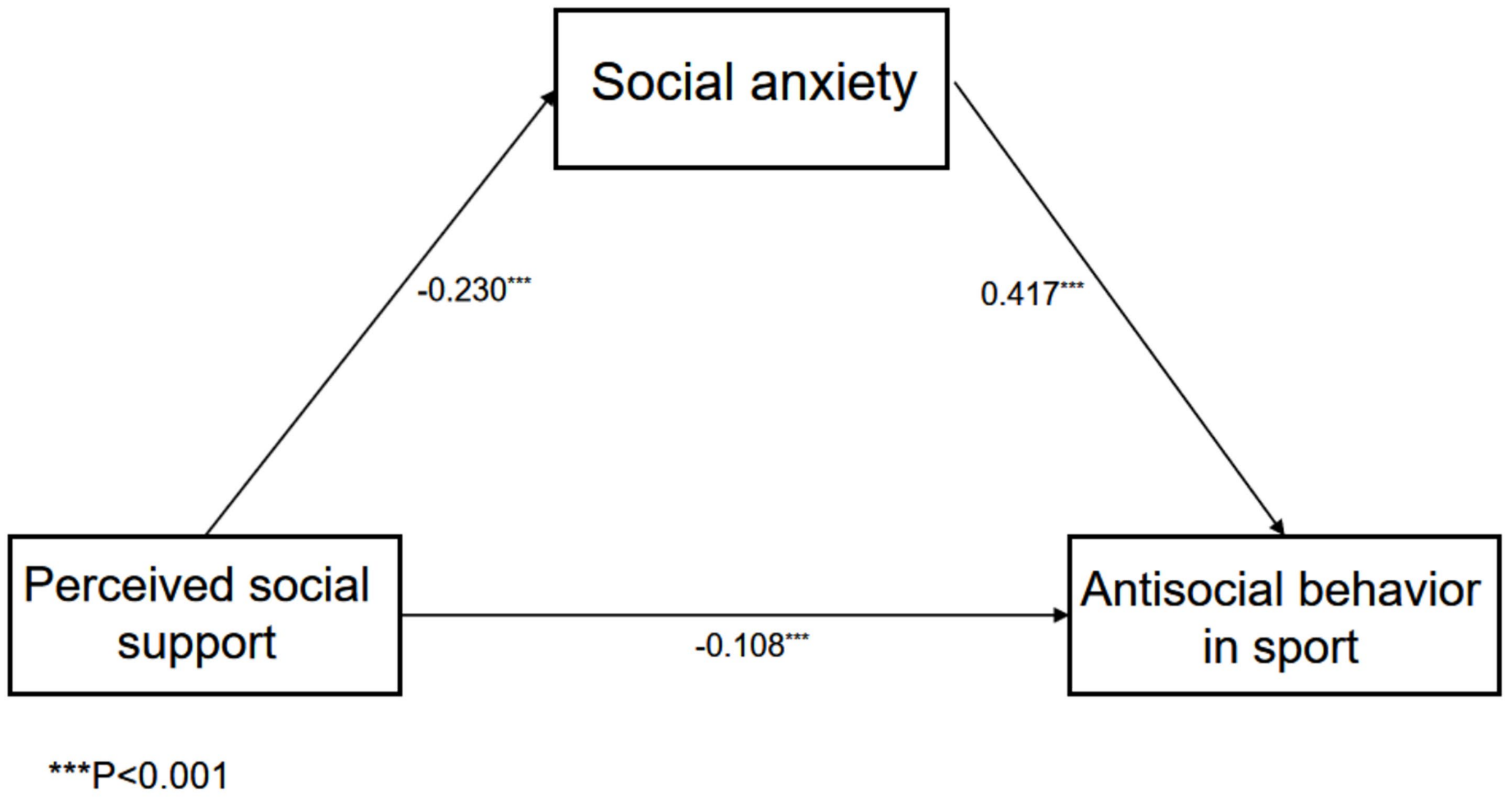
The mediation model of social anxiety.

The mediation analysis was conducted within Mplus 8.3. The corresponding results are presented in Figure 4. The analysis revealed that: perceived social support negatively predicts antisocial behavior in sports (*β* = −0.108, *p* < 0.01) and negatively predicts social anxiety (*β* = −0.230, *p* < 0.01). Furthermore, social anxiety positively predicts antisocial behavior in sports (*β* = 0.417, *p* < 0.01). These findings ‌support Hypothesis 1. In accordance with the requirements for detecting mediation effects, the Bootstrap method with 5,000 iterations (Jeremy et al., 2010), as detailed in Table 2. The confidence interval at 95% excluded 0, the results indicate that social anxiety mediates the association between perceived social support and antisocial behavior among athletes, accounting for an effect size of 47%. Thus, Hypothesis 2 is also supported: The association between social support and antisocial behavior in sports is indirectly influenced through social anxiety.

**Table 2:** Social anxiety mediation effect test in model.

Path	*β*	S. E.	Est./S. E.	*p*	Bootstrapped 95% CI		Relative effects
					Lower	Upper	
Direct effect	−0.108	0.014	−7.751	0.000	−0.138	−0.157	53%
Mediation effect	−0.096	0.020	−4.686	0.000	−0.141	−0.062	47%
Total effect	−0.204	0.028	−7.413	0.000	−0.262	−0.157	

### 3.4 Psychological resilience moderation effect

#### 3.4.1 The relationship between social support and social anxiety is moderated by psychological resilience

**Figure 5:**
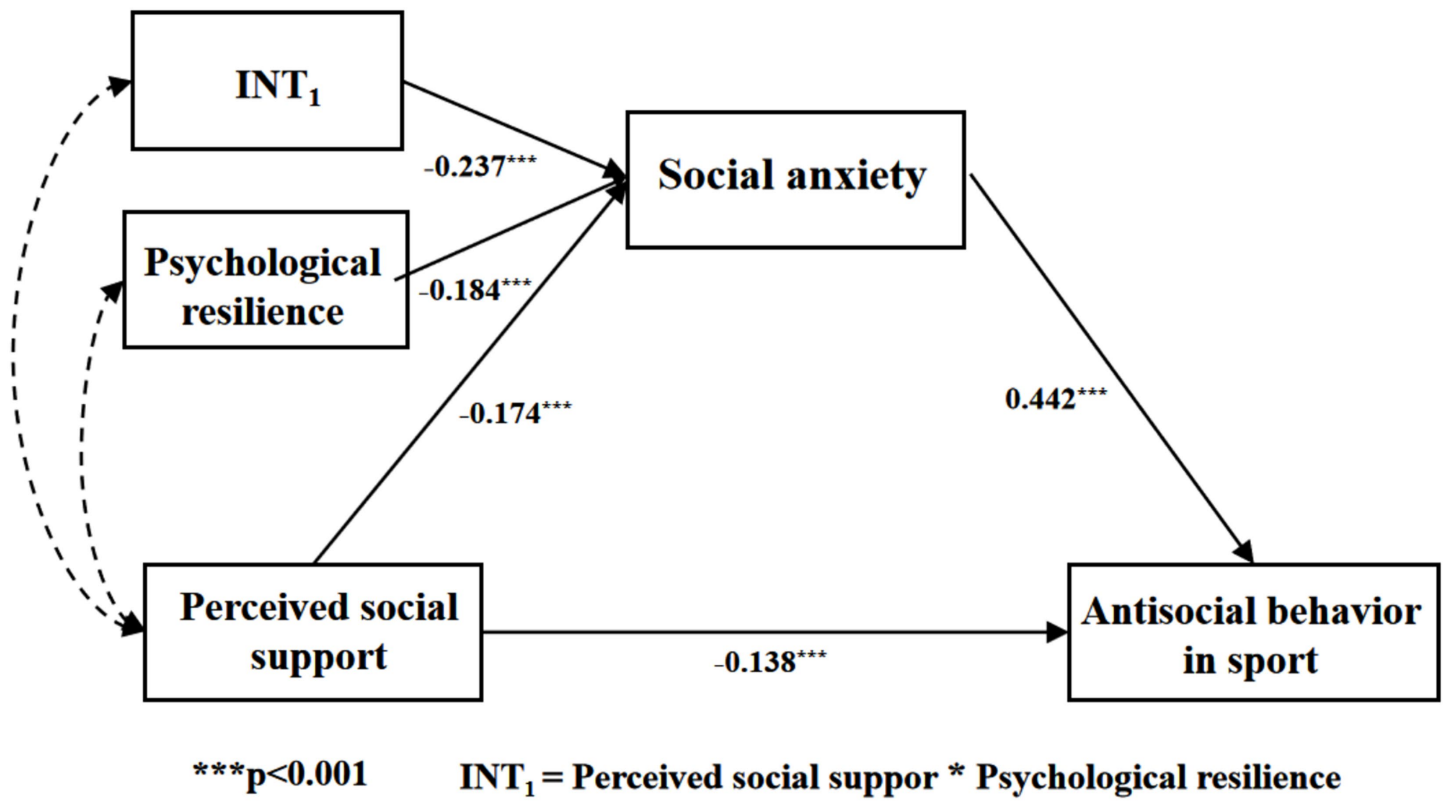
The moderated mediation model of psychological resilience (INT_1_) on social anxiety by social support.

As presented in Table 3, psychological resilience exhibited a significant negative relationship with social anxiety (*β* = −0.184, *p* < 0.001). Additionally, the interaction term (INT_1_) which combines perceived social support with psychological resilience demonstrated Significantly reduced social anxiety, reflected by a coefficient of *β* = −0.237 (*p* < 0.01). Figure 5 illustrates the moderating role of psychological resilience in the association between perceived social support and social anxiety. The regression coefficient of the interaction term (VI × VMod) is significant, indicating the presence of a moderating effect (Pier-Olivier, 2018). Furthermore, the Bootstrap method (5,000 iterations) was employed to assess the moderated model. The 95% confidence interval for the interaction term between perceived social support and psychological resilience excluded 0 (see Table 3). This indicates that psychological resilience moderates the relationship between perceived social support and social anxiety (Jeremy et al., 2010). Hypothesis 3 is thus confirmed.

**Table 3:** The moderating coefficient of psychological resilience (INT_1_) on social anxiety by social support.

Path	*β*	*E*.	Est./S. E.	*p*	Bootstrapped 95% CI	
					Lower	Upper
W	−0.184	0.034	−5.011	0.000	−0.784	−0.342
INT_1_	−0.237	0.046	−4.613	0.000	−1.025	−0.412

W = Psychological resilience.

INT_1_ = Perceived social support * Psychological resilience.

**Figure 6:**
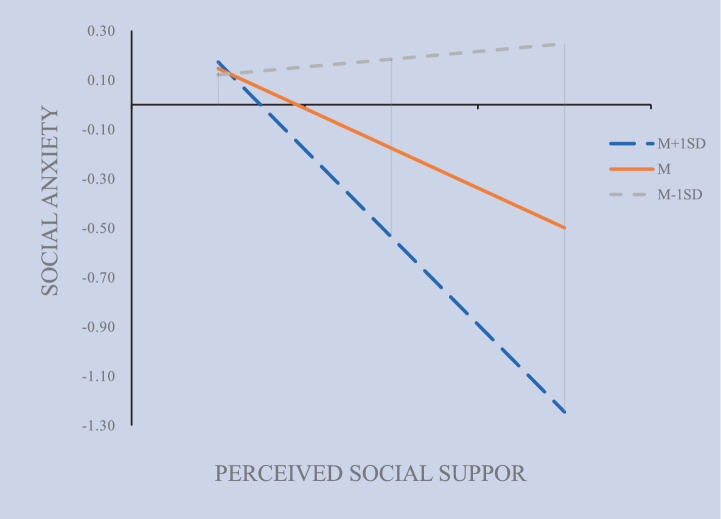
The moderation of psychological resilience (INT_1_) on social anxiety by social support.

As shown in Table 4, Psychological resilience significantly moderated the relationship between perceived social support and social anxiety. At low levels of psychological resilience, (*β* = 0.072), the statistical significance test does not reach the significant level (*p* > 0.05), whereas the mediating (*β =* −0.224) and moderating effects at high levels (*β =* −0.520) are significant (*p* < 0.05). Figure 6 presents the Johnson-Neyman interval diagram of psychological resilience (INT_1_) at both low (M − 1 standard deviation) and high (M + 1 standard deviation) levels. The diagram employs a regression line to depict the relationship between the predictor and moderating variables, with the effect variable regressed onto the moderating variable (Hua, 2020). The findings indicate that psychological resilience moderates the effect of social support on social anxiety, such that higher levels of resilience strengthen this effect.

**Table 4:** Psychological resilience (INT_1_) ± 1 standard deviation coefficient.

Path	*β*	S. E.	Est./S. E.	*p*	Bootstrapped 95% CI	
					Lower	Upper
M − 1SD	0.072	0.090	−5.486	0.427	−0.080	0.276
M	−0.224	0.052	−4.339	0.000	−0.338	−0.137
M + 1SD	−0.520	0.095	0.794	0.000	−0.709	−0.347

#### 3.4.2 Psychological resilience moderates the relationship between social anxiety and prosocial behavior in sports

**Figure 7:**
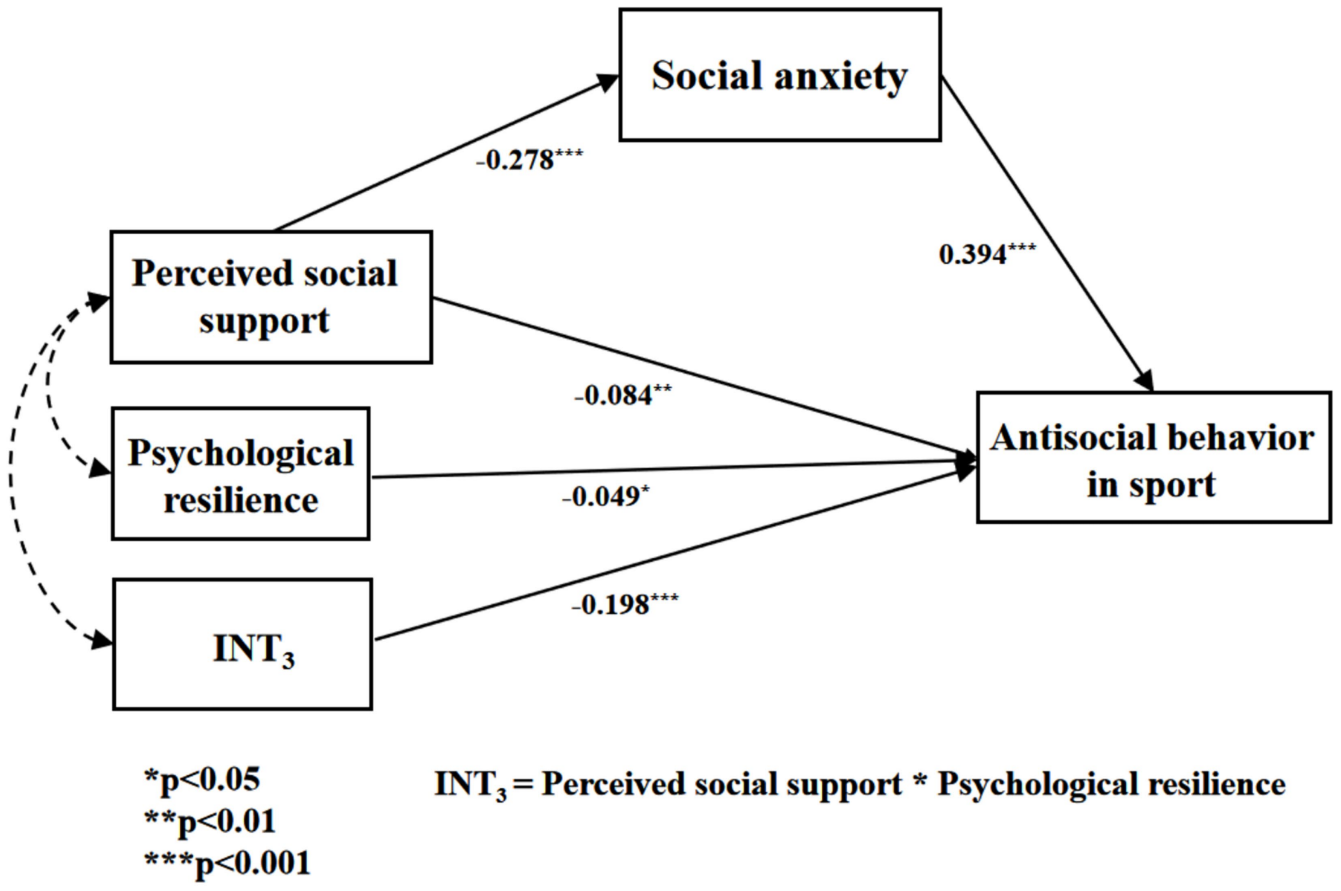
The moderated mediation model of psychological resilience (INT_2_) on antisocial behavior in sports by social anxiety.

Results in Table 5 reveal a negative correlation between psychological resilience and antisocial behavior in sports (*β* = −0.143, *p* < 0.05), Furthermore, the interaction term (INT_2_)—combining social anxiety and psychological resilience—exhibited a significant positive effect on antisocial behavior in sport (*β* = 0.267, *p* < 0.001), this indicates that the variable of psychological resilience (INT_2_) serves as a moderator (Pier-Olivier, 2018). Using psychological resilience as the moderating influence, a structural model linking social anxiety with antisocial behavior in sports was created (Figure 7). In addition, the Bootstrap approach (5,000 iterations) was utilized to analyze the moderated model. The 95% confidence interval for the interaction term concerning perceived social support and psychological resilience excludes 0 (Table 5), confirming that psychological resilience significantly alters the relationship between social anxiety and antisocial behavior (Jeremy et al., 2010). Therefore, Hypothesis 4 is validated.

**Table 5:** The moderating coefficient of psychological resilience (INT_2_) on antisocial behavior in sports by social anxiety.

Path	*β*	S. E.	Est./S. E.	*p*	Bootstrapped 95%CI	
					Lower	Upper
W	−0.143	0.072	−10.983	0.047	−0.281	−0.003
INT_2_	0.267	0.047	5.715	0.000	0.168	0.351

W = Psychological resilience.

INT_2_ = social anxiety * Psychological resilience.

**Figure 8:**
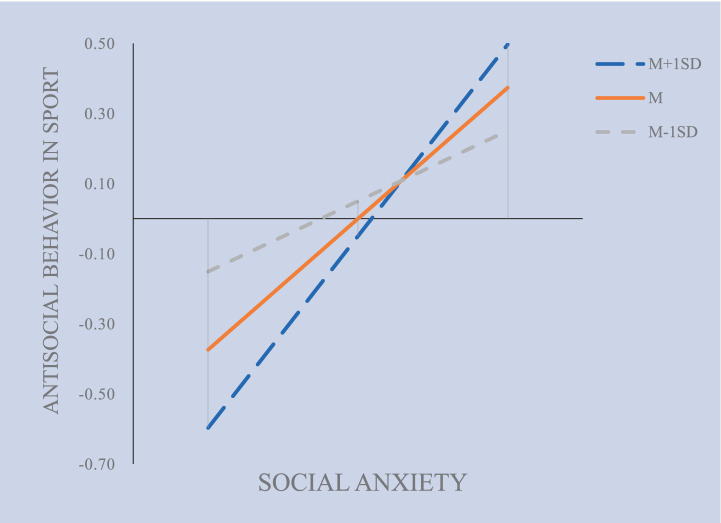
The moderation of psychological resilience (INT_2_) on antisocial behavior in sports by social anxiety.

As shown in Table 6, indicates that the moderating effect of psychological resilience at low (*β =* −0.059) and the mediating effect (*β = −*0.079), as well as the moderating effect at high (*β = −*0.099), are significant (*p* < 0.05). Figure 8 presents the Johnson-Neyman interval diagram of psychological resilience (INT_2_) at Low (−1 standard deviation) and High (+1 standard deviation) levels. The diagram employs a regression line to depict the relationship between the predictor and moderating variables, with the effect variable regressed onto the moderating variable (Hua, 2020). The results reveal a positive predictive relationship between social anxiety and antisocial behavior in sports. As social anxiety escalates, the influence of psychological resilience plays a crucial role in reducing the antisocial behavior related to sports that arises from this anxiety. Nevertheless, it is significant to highlight that the impact of psychological resilience as a moderator progressively lessens with the increase of social anxiety.

**Table 6:** The moderating effect of psychological resilience (INT_2_) at different levels.

Path	*β*	S. E.	Est./S. E.	*p*	Bootstrapped 95%CI	
					Lower	Upper
M − 1SD	−0.059	0.013	−4.501	0.000	−0.090	−0.038
M	−0.079	0.016	−5.004	0.000	−0.114	−0.053
M + 1SD	−0.099	0.020	−5.045	0.000	−0.142	−0.066

#### 3.4.3 Psychological resilience moderates the relationship between social support and antisocial behavior in sports

**Figure 9:**
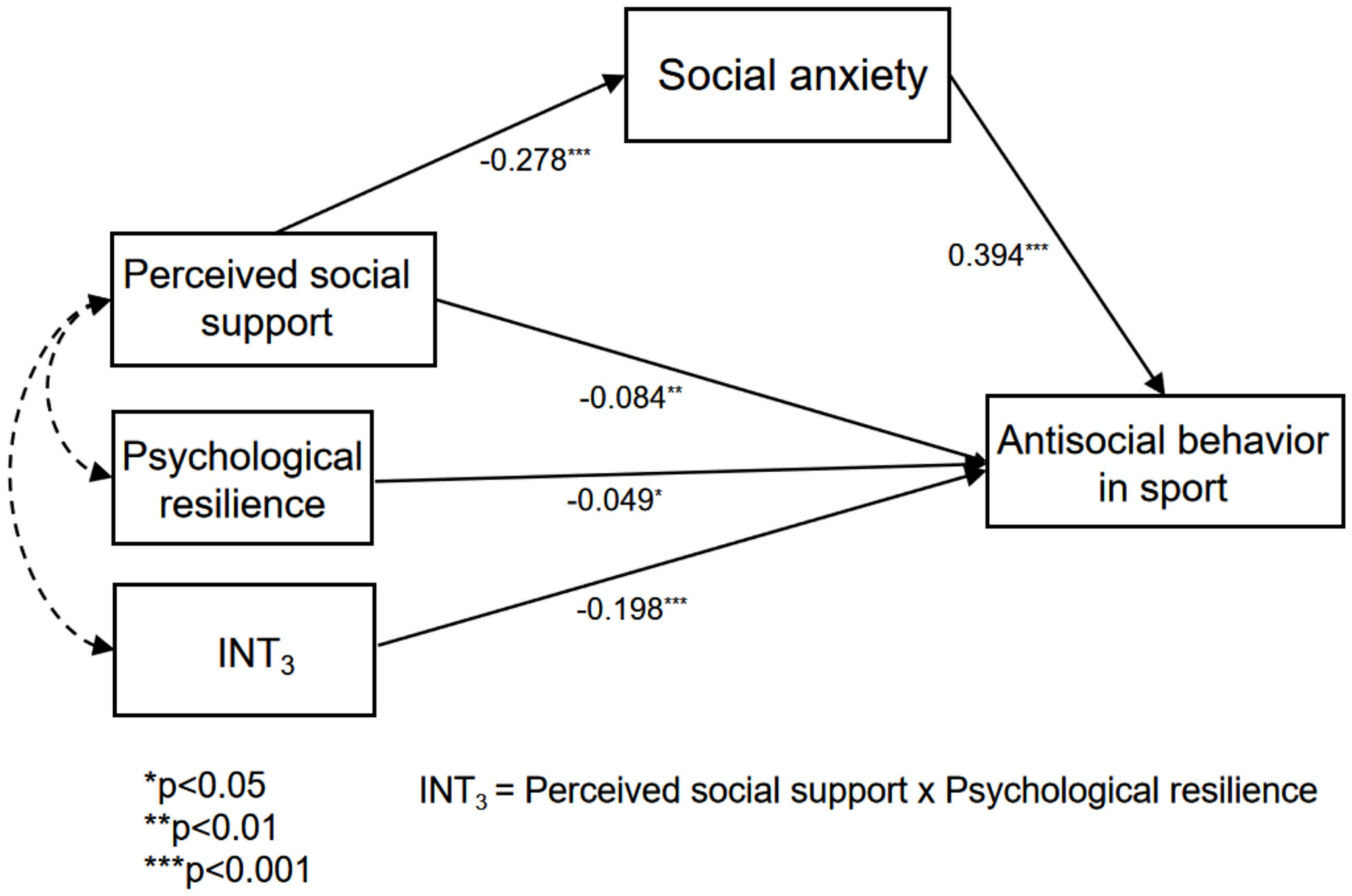
The moderated mediation model of psychological resilience (INT_3_) on antisocial behavior in sports by social support.

As indicated in Table 7, psychological resilience demonstrates a significant negative correlation with antisocial behavior in sports (*β* = −0.049, *p* < 0.05). Importantly, the interaction term (INT_3_)—combining perceived social support and psychological resilience—exhibits a significant negative predictive effect (*β* = −0.198, *p* < 0.001), confirming that psychological resilience moderates the relationship between social support and antisocial behavior (Pier-Olivier, 2018). To validate these outcomes, the Bootstrap method was utilized with 5,000 iterations to evaluate the moderated model. The 95% confidence interval for the interaction term involving perceived social support and psychological resilience does not encompass 0 (Table 7), further validating that psychological resilience serves a moderating function in the connection between perceived social support and antisocial behavior in sports (Jeremy et al., 2010). Consequently, a model depicting the moderating influence of psychological resilience on the interplay between social support and antisocial behavior in sports is illustrated in Figure 9. Therefore, Hypothesis 5 is substantiated.

**Table 7:** The moderating coefficient of psychological resilience (INT_3_) on antisocial behavior in sports by social support.

Path	*β*	S. E.	Est./S. E.	*p*	Bootstrapped 95%CI	
					Lower	Upper
W	−0.049	0.025	−1.982	0.047	−0.097	−0.002
INT_3_	−0.198	0.030	−6.695	0.000	−0.7258	−0.143

W = Psychological resilience.

INT_3_ = perceived social support * Psychological resilience.

**Figure 10:**
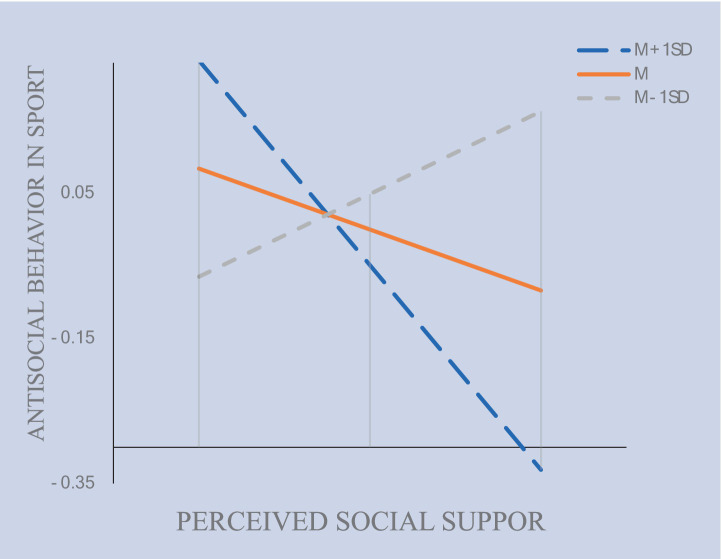
The moderation of psychological resilience (INT_3_) on antisocial behavior in sports by social support.

Table 8 demonstrates that the moderating effect of psychological resilience at low (*β = −*0.074), as well as the mediating effect (*β =* −0.312) and the moderating effect at high (*β =* 0.190), are statistically significant (*p* < 0.05). Figure 10 presents the Johnson-Neyman interval diagram of psychological resilience (INT_3_) at Low (−1 standard deviation) and High (+1 standard deviation) levels. The diagram employs a regression line to depict the relationship between the predictor and moderating variables, with the effect variable regressed onto the moderating variable (Hua, 2020). Social support is associated with a decrease in antisocial behavior in sports. Additionally, psychological resilience plays a moderating role by enhancing the influence of social support on antisocial behaviors in sports, effectively reducing their frequency.

**Table 8:** The moderating effect of psychological resilience (INT_3_) at different levels.

Path	*β*	S. E.	Est./S. E.	*p*	Bootstrapped 95%CI	
					Lower	Upper
M − 1SD	−0.074	0.023	−3.214	0.001	−0.123	−0.037
M	−0.312	0.064	−4.902	0.000	−0.451	−0.203
M + 1SD	0.190	0.068	2.773	0.006	0.067	0.341

## 4 Discussion

### 4.1 Social support negatively predicts antisocial social behavior in sports

The results of this research reveal a notable correlation between perceived social support and antisocial behavior in sports. This aligns with prior research (Liu et al., 2021). The multifaceted conceptual framework of social support, as highlighted by Xiaolin and Rajiv (2021), argues that perceived social support functions as a vital resource—both psychological and social—offering crucial environmental assistance to individuals. College students take up sports, low levels of perceived social support among college students can lead to feelings of alienation from peers and the environment, difficulties in team integration, and an inability to express negative emotions stemming from intrusive symptoms. Consequently, individuals may be more prone to express anger and fear through antisocial behaviors in sports, such as aggression and rule violations. Positive interpersonal relationships have been shown to effectively mitigate the negative emotions associated with intrusive symptoms (Xiaolin and Rajiv, 2021), and reducing antisocial behaviors through prosocial alternatives (e.g., teammate encouragement, constructive feedback) This evidence underscores the dual protective role of social support—both as a direct suppressor of antisocial behavior and as a mediator that enhances emotional regulation in sports contexts (Brechwald and Prinstein, 2011).

### 4.2 The mediating role of social anxiety

Our findings reveal that social anxiety serves as a mediating factor in how perceived social support relates to antisocial behaviors in sports. This aligns with prior research conducted in this field (Beauchamp, 2015), showing that perceived social support has a negative effect on social anxiety in college students. Furthermore, research has demonstrated that sports enhance individuals’ emotional management skills, with interpersonal interactions serving as a primary catalyst for emotional changes (Dianne, 1992). In a sporting context, positive interactions between college students and their teammates, as well as within the group as a whole, facilitate the acquisition of psychological support. The sense of being valued and respected by teammates or peers has been shown to enhance self-esteem and self-confidence. This improved self-perception encourages active and proactive behavior in social situations, while simultaneously reducing the levels of social interaction apprehension experienced in sports (Forsdyke et al., 2022). Study also found that social anxiety negatively predicts prosocial behavior in sports, which aligns with previous research (Danae et al., 2023). Individuals with high levels of social anxiety often experience shyness, low self-worth, and social avoidance. People who show significant anxiety are often accompanied by obvious shyness, lack of self-identity and a tendency to avoid social interaction (Jana et al., 2022). This phenomenon has been shown to exacerbate the occurrence of antisocial behavior in sports. To extricate themselves from this interpersonal predicament, college students often display an imbalance between organizational and interpersonal orientation, leading to irrational antisocial behavior in sports (Lu et al., 2022). Within the theoretical framework of social interactionism, as a form of collective interaction, physical activity helps to enhance interpersonal communication and improve the efficiency of social mutual assistance (Yu et al., 2023). Consequently, in the context of educational activities, educators actively cultivate an environment of collective cooperation. Through practical learning experiences, students acquire the ability to communicate and collaborate effectively, enhance their interpersonal skills, alleviate social anxiety during physical activities, and develop prosocial sports behaviors, such as actively assisting and encouraging teammates (Yu et al., 2023). The positive culture that emerges from these activities has been shown to elevate students’ levels of social support, ensuring that when they subsequently engage in sports, they experience a heightened sense of social support, communicate more actively with others, and reduce antisocial behaviors in sports.

### 4.3 The moderating effect of psychological resilience

This study’s findings indicate that psychological resilience serves as a moderator in the relationships linking social support to social anxiety, social anxiety to antisocial behavior in sports, and social support to antisocial behavior in sports.

First, perceived social support and social anxiety are moderated by psychological resilience, aligning with prior research (Yi et al., 2023). The social support buffering hypothesis (Peggy, 2011) and the psychological resilience framework (Allison et al., 2023) suggest that psychological resilience is thematically linked to individuals’ perceived social support. In regarding to external stress and conflict, Psychological resilience serves as a buffer, mitigating the adverse effects of these tensions on mental health. The regulatory effect of psychological elasticity can work through various interconnection paths: (1) Cognitive Reappraisal: Resilient individuals demonstrate an enhanced capacity for cognitive restructuring (Peter et al., 2019), allowing them to reinterpret social stressors (e.g., criticism from teammates or coaches) as challenges rather than threats. This shift reduces fears of social evaluation, a core component of social anxiety (Hofmann, 2010). (2) Resource Activation: High-resilience athletes utilize existing social support more effectively by proactively seeking guidance during stressful situations (Swati and Updesh, 2016). This counters the withdrawal tendencies of social anxiety, disrupting avoidance-distress cycles. (3) Neurobiological Regulation: Psychological resilience correlates with reduced amygdala reactivity to social threats and enhanced prefrontal cortex modulation (Kang and Qian Hui, 2021), potentially dampening anxiety-related physiological arousal. While prior research indicates that psychological resilience influences college students’ physical activity and social anxiety (Xin et al., 2024), our study clarifies its context-specific moderating role in athletic settings. This distinction underscores the importance of situational specificity: in high-stakes sports environments, resilience may act as a dynamic buffer rather than merely serving as a conduit for support. Importantly, our findings indicate that psychological resilience weakens the link between social support and anxiety, suggesting that this buffering effect is particularly critical in competitive settings where social evaluation is prevalent. Interventions focused on enhancing psychological resilience should integrate cognitive-behavioral training (e.g., reframing negative self-talk) and mindfulness practices into athletic programs to strengthen resilience (Nick and Robin, 2008). Additionally, increasing social support between coaches and players is essential; coaches should cultivate ‘autonomy-supportive’ environments (Noam Eliezer, 2020) that empower athletes to seek help, aligning with the proactive coping styles of high-resilience individuals. Regarding early identification and screening, it is advisable to assess athletes with low social support for resilience deficits to implement targeted prevention strategies before social anxiety develops.

Second, this study’s findings support prior research (Ahmad Ali et al., 2023) by demonstrating that psychological resilience moderates the association between social fear and antisocial behavior in sport. This finding supports self-control theory (Chris and Wanja, 2015), which postulates that psychological resilience serves as a dynamic coping resource that mitigates the negative effects of social anxiety on antisocial behavior in sports through emotional regulation mechanisms and cognitive reappraisal strategies. (1) Regarding emotional regulation mechanisms, individuals with high resilience exhibit greater emotional stability under social anxiety (Gabriela et al., 2024). Elevated resilience weakens the predictive power of social anxiety on antisocial behavior, inhibiting emotional dysregulation and preventing anxiety-driven aggression. (2) Psychological resilience acts to inhibit emotional loss of control induced by anxiety, thereby preventing its escalation into aggressive behavior. In terms of cognitive reappraisal strategies, athletes with substantial psychological resilience are more inclined to adopt adaptive cognitive frameworks (Michele and Barbara, 2004). (3) Confirmatory factor analysis revealed that the direct effect of the “goal focus” dimension on antisocial behavior was most pronounced within the psychological resilience scale, thereby confirming the deconstructive effect of goal-oriented thinking on anxiety. In contrast to existing research, particularly that of Donna et al. (2019), which indicated that psychological resilience influences antisocial behavior in both team and individual sports, this study elucidates the diverse moderating effects of psychological toughness in sports behavior performance. Resilience emerges as a critical protective factor between social anxiety and antisocial behavior, offering a new theoretical lens for sports psychology. Future Directions. Research should explore: (1) psychological resilience training (e.g., CBT, mindfulness) and (2) AI-driven interventions for scalable, personalized support.

Third, this study demonstrates that psychological resilience significantly moderates the relationship between social support and antisocial behavior in sport. This result is consistent with previous tests (Ru et al., 2024). This finding aligns with and extends contemporary theoretical frameworks of psychological resilience, which emphasize its multidimensional capacity to buffer adverse psychological and behavioral outcomes in high-pressure environments (Songli et al., 2021). (1) When athletes perceive inadequate social support (e.g., lack of trust from coaches or peer cohesion), those with high psychological resilience demonstrate a greater capacity to mitigate resultant stressors. As evidenced in studies of individual-sport athletes (e.g., MMA), psychological resilience cultivates traits such as self-regulation, emotional control, and perseverance (Zachary et al., 2021). This reframing reduces the likelihood of resorting to antisocial behaviors (e.g., aggression, rule violations) as maladaptive coping responses. (2) Our results support the resilience-compensation hypothesis: high psychological resilience compensates for low social support by activating internal resources (Fred et al., 2006). Psychological resilience moderates the pathway from social support to antisocial behavior by strengthening moral disengagement thresholds. Athletes with high psychological resilience exhibit heightened integrity and composure under stress, traits that are directly associated with reduced antisocial conduct (Mustafa and David, 2013). This aligns with the role of psychological resilience in promoting adherence to ethical norms through self-determination and goal congruence (Aijing et al., 2020). (3) Our findings expand purely linear models of social support and behavior by emphasizing psychological resilience as a contextual catalyst. While traditional frameworks prioritize individual attributes such as control and commitment, our results advocate for the integration of interpersonal dimensions (Amy and Michael, 2021). In team sports, the moderating role of psychological resilience manifests through shared resilience, where athletes with high psychological resilience reinforce group norms that discourage antisocial behaviors, even when support structures weaken (Anouk et al., 2018). This supports observations that psychological resilience in team contexts is contingent upon relational dynamics.

Based on the findings that social support reduces antisocial behavior in sports through decreasing social anxiety while psychological resilience serves as a crucial moderator in these relationships, we recommend implementing an integrated intervention approach combining structural support systems and skill-building components. The proposed “Athlete-Coach-Psychological Consultant” (Michael et al., 2024) tripartite support system should work synergistically with Peer Support Leader programs to enhance social networks, while simultaneously incorporating resilience-building exercises such as video replay analysis and simulated competition scenarios into regular training to normalize competitive stress. Daily micro-interventions including mindfulness exercises and cognitive reframing drills (Freddie, 2022) should be embedded to sustain resilience activation, complemented by immediate emotion-regulation tools like controlled breathing techniques to address anxiety in competitive moments. This comprehensive strategy – combining systemic support structures, peer-mediated social adaptation, scenario-based resilience training, embedded psychological skill development, and real-time regulatory techniques – aims to create a sustained buffering effect against antisocial behaviors in sport by simultaneously strengthening protective factors (social support and psychological resilience) while mitigating risk factors (social anxiety).

## 5 Limitations

This research adds to the current body of knowledge regarding social support and behaviors in sports contexts. Nonetheless, it does have certain limitations. Firstly, the use of a cross-sectional design limits the capacity to determine causal links among the variables. Future studies ought to explore longitudinal intervention experiments. Secondly, the research primarily examines how social anxiety and psychological resilience impact the relationship between social support and social behavior in sports among college students, which may neglect other potentially influential factors, such as students’ motivation for engaging in sports and their personality characteristics, both of which deserve further exploration. Lastly, future research should aim to broaden the sample size to improve the external validity and generalizability of the results. Social support can be classified into actual social support and perceived social support, which could further inform studies examining the connection between actual social support and antisocial behavior in athletic settings.

## 6 Conclusion

This study demonstrates that social support effectively mitigates antisocial behaviors in sports settings, with social anxiety acting as a mediating variable. Notably, psychological resilience emerges as a significant moderator across three critical pathways: the association between social support and social anxiety, the relationship between social anxiety and antisocial behaviors in sport, and the relationship between social support and antisocial behaviors in sport. Based on these empirical insights, we propose a comprehensive intervention framework featuring: (1) an integrated “athlete-coach-mental coach” support network to enhance perceived social support; (2) evidence-based psychological resilience training incorporating match review protocols and stress inoculation techniques; and (3) targeted social anxiety reduction through mindfulness-based interventions and cognitive restructuring programs. These multilayered strategies collectively form a robust protective mechanism against antisocial conduct in athletic contexts.

## Data Availability

The raw data supporting the conclusions of this article will be made available by the authors, without undue reservation.
